# Meetings of Societies

**Published:** 1932

**Authors:** 


### Meetings of Societies

### Bristol Medico-Chirurgical Society

SESSION 1931-32.

The Annual General Meeting was held in the Physiological Lecture Theatre of the University on Wednesday, 12th October, 1932, at 8.30 p.m.

Dr. J. R. Charles was in the Chair, one hundred and five members being present.

The minutes of the previous meeting were read and confirmed.

One candidate, whose name was proposed and seconded at the previous meeting was elected to the membership of the Society.

Five candidates were proposed for election to the membership of the Society.

The President, Dr. J. R. Charles, then resigned the Chair to his successor, Mr. E. W. Hey Groves.

Dr. P. Watson-Williams proposed a vote of thanks to Dr. J. R. Charles for his services during the past year ; this was seconded by Professor Rayner and carried with acclamation, and Dr. Charles replied.

Mr. E. W. Hey Groves then gave his inaugural address entitled : **"A Surgical Adventure."**

Mr. C. F. Walters proposed a vote of thanks to the President for his addiess ; this was seconded by Mr. Duncan Wood and carried unanimously.

The Hon. Secretary and Hon. Treasurer then gave their reports, and the Editor of the *Journal,* Professor J. A. Nixon, then gave his report and spoke of the history of the *Journal.*

Dr. Elwin Harris (senior) was elected to the office of President-Elect on the proposition of Professor A. Rendle Short, seconded by Dr. Carey Coombs.

### BRISTOL MEDICO-CHIRURGICAL SOCIETY.

### INCOME AND EXPENDITURE ACCOUNT for the year ended 30th September, 1932.

### INCOME.

| | £ | s. | d. | £ | s. | d. |
|---|---|---|---|---|---|---|
| Members' Subscriptions .. | 228 | 18 | 0 | | | |
| Do., outstanding for 1931–1932 .. .. .. .. .. | 59 | 17 | 0 | | | |
| | | | | 288 | 15 | 0 |
| Interest on Bank Deposits .. .. | | | | 3 | 9 | 6 |
| Interest on £500 4 per cent. Consolidated Stock, less tax .. .. | | | | 14 | 15 | 0 |
| Balance, being excess of Expenditure over Income for the year carried to Balance Sheet .. .. .. | | | | 6 | 4 | 0 |
| | | | | £313 | 3 | 6 |

### EXPENDITURE.

| | £ | s. | d. | £ | s. | d. | £ | s. | d. |
|---|---|---|---|---|---|---|---|---|---|
| Grant to *The Bristol Medico-Chirurgical Journal* .. .. .. .. | | | | | | | 250 | 1 | 4 |
| Librarian—Honorarium .. .. .. | | | | | | | 15 | 0 | 0 |
| Miscellaneous Expenses— | | | | | | | | | |
| Goodway–Refreshments | | | | 16 | 16 | 0 | | | |
| Postages and Sundries | | | | 9 | 9 | 11 | | | |
| Printing and Stationery | | | | 9 | 7 | 0 | | | |
| Audit Fee .. .. .. .. | | | | 2 | 2 | 0 | | | |
| Lantern and Cinematograph at Meetings .. | | | | 4 | 4 | 0 | | | |
| University Marshal .. | | | | 1 | 0 | 0 | | | |
| Lewis's Medical and Scientific Library— | | | | | | | | | |
| Subscription | 4 | 4 | 0 | | | | | | |
| Postages .. | 0 | 19 | 3 | | | | | | |
| | | | | 5 | 3 | 3 | | | |
| | | | | | | | 48 | 2 | 2 |
| | | | | | | | £313 | 3 | 6 |

### BALANCE SHEET as at 30th September, 1932.

### LIABILITIES AND CAPITAL.

| | £ | s. | d. | £ | s. | d. |
|---|---|---|---|---|---|---|
| Sundry Creditors .. .. | | | | 105 | 18 | 2 |
| Capital as at 30th Sept., 1931 .. .. .. .. .. | 686 | 5 | 6 | | | |
| *Less* Deficit on Income and Expenditure Account as above .. | 6 | 4 | 0 | | | |
| | | | | 680 | 1 | 6 |
| | | | | £785 | 19 | 8 |

### ASSETS.

| | £ | s. | d. | £ | s. | d. |
|---|---|---|---|---|---|---|
| Cash at Bank— | | | | | | |
| Current Account .. .. | 127 | 17 | 2 | | | |
| Deposit Account .. .. | 160 | 16 | 6 | | | |
| | | | | 288 | 13 | 8 |
| £500 4 per cent. Consolidated Stock at cost .. .. .. .. .. .. | | | | 437 | 9 | 0 |
| Sundry Debtors— Members' Subscriptions for 1931–1932 outstanding .. | | | | 59 | 17 | 0 |
| | | | | £785 | 19 | 8 |

Audited and found correct,

H. E. KEELER,

*Chartered Accountant,*

*Auditor.*

16-18 Clare Street, Bristol,

*10th October,* 1932.

Mr. Carwardine proposed and Mr. G. B. Bush seconded the re-election of Mr. H. L. Shepherd as Hon. Secretary.

Dr. Corfield proposed and Dr. Herbert Rogers seconded the re-election of Mr. A. E. Iles as Hon. Treasurer.

Dr. Geo. Parker was unanimously re-elected as Hon. Medical Librarian on the proposal of Mr. Chitty, seconded by Dr. Dix.

Dr. G. Parker, Mr. Hey Groves and Dr. Symes were elected to serve on the University Medical Library Sub-Committee, on the proposal of Dr. Carleton, seconded by Dr. Perry.

Mr. A. E. Iles proposed and Dr. Richard Clarke seconded that the following addendum should be made to Law 7: "That the name of any member whose subscription is three years in arrears shall be removed from the list of members."

The Society's Annual Dinner was then discussed, and it was proposed by Mr. C. F. Walters and seconded by Dr. Richard Clarke that this should take place in March, 1933.

---

The second meeting of the session was held in the Physiological Lecture Theatre of the University on Wednesday, 9th November, 1932, at 8.30 p.m. Mr. E. W. HEY GROVES in the Chair, and eighty-six members present.

The minutes of the previous meeting were read and confirmed.

Dr. WANSBROUGH and Dr. HERBERT ROGERS showed respectively X-rays and specimens.

The PRESIDENT proposed and it was carried unanimously that Dr. J. A. Nixon should be elected as the Society's representative to serve on the Conjoint Committee of Epsom College.

Dr. MACDONALD CRITCHLEY read a paper on "**The Effects of Lightning**" (reported on page 285), which was afterwards discussed.

